# Clinical Diagnosis and Management of Mucosal Leishmaniasis in the Context of a Global Pandemic: A Case Report

**DOI:** 10.7759/cureus.30586

**Published:** 2022-10-22

**Authors:** Alessandro Carotenuto, Grant D Albers, Richard Hankins, Katie Geelan-Hansen

**Affiliations:** 1 Otolaryngology - Head and Neck Surgery, University of Nebraska Medical Center, Omaha, USA; 2 Internal Medicine, University of Nebraska Medical Center, Omaha, USA

**Keywords:** cutaneous leishmaniasis, miltefosine, sodium stibogluconate, meglumine antimonate, mucosal leishmaniasis, leishmania vianna

## Abstract

Mucosal leishmaniasis (ML) is a rare metastatic complication of Leishmania infection. It has a high potential for destructive and disfiguring complications, namely destruction of nasal architecture and airway compromise. ML is difficult to treat for a variety of reasons, including variable antimicrobial resistance rates between species, as well as between endemic areas geographically. There are several treatment options available, which are discussed here. In the majority of cases, a nuanced approach to treatment is required based on speciation and geography. Importantly, the treatment of ML requires a multi-disciplinary approach. We present a patient with a history of cutaneous leishmaniasis who presented with signs and symptoms concerning ML, but due to the COVID-19 global pandemic diagnostic testing was not possible, was treated empirically under clinical suspicion of ML with good results.

## Introduction

Leishmaniasis is a complex of diseases caused by the Protozoa genus Leishmania. This vector-borne illness is transmitted through the bite of an infected phlebotomine sandfly. Only the female sandfly feeds on mammals because it requires blood meals for proper egg development [1]. Clinical manifestations exist in three varieties: Cutaneous, Mucosal, and visceral [2]. Cutaneous leishmaniasis (CL) is considered an endemic disease, spanning more than five continents, 98 countries, and 350 million people with an estimated annual incidence of 400,000 [3,4]. Leishmaniasis is grossly divided into two geographical subsets, “New World” and “Old World.” The term “New World” leishmaniasis refers to endemic infections of South and Central America and is attributable to the species L. mexicana and the Vianna complex (L.V. braziliensis, L.V. guyanensis, and L.V. panamensis). in Contrast, “Old World” leishmaniasis refers to endemic infections of the Middle East and are attributable to L. major and L. tropica. Collectively, these "New World" species cause “American Tegumentary Leishmaniasis” (ATL), which includes cutaneous and mucocutaneous leishmaniasis. ATL is one of 17 neglected tropical diseases. The neglect is largely due to the diverse clinical presentation and varying treatment response that makes ATL extremely difficult to diagnose and treat [2,3,5].

Mucosal leishmaniasis (ML), also known as espundia, is most commonly seen in South and Central America and is caused by the Vianna subgenus listed above. CL and ML are endemic to regions of Bolivia, Peru, Brazil, and the Guianan Ecoregion Complex, which covers Guyana, Suriname, French Guiana, parts of Venezuela, and the northern Amazon basin. ML is considered a metastatic sequela of a CL infection that has spread to the naso-oropharyngeal/laryngeal mucosa. The dissemination of the parasite occurs in less than 5% of CL cases, and mucosal lesions can appear even years after initial cutaneous lesions are present [2,3,6]. ML most commonly presents as ulcerative lesions of the upper aerodigestive tract. The mucosa of the nasal cavity, oral cavity, oropharynx, and larynx are often affected, with the nasal septum and mouth being the most common sites [7,8]. These findings are preceded by symptoms of nasal obstruction, epistaxis, chronic dry cough, hoarseness, and/or odynophagia. An additional concern is the possibility of significant facial disfigurement secondary to nasal cartilage erosion.

Treating ML requires a multidisciplinary approach involving infectious disease, otolaryngology, and facial plastic surgery departments. Assistance from the Centers for Disease Control and Prevention (CDC) reference laboratories must also be utilized to ensure correct diagnosis and prevent mismanagement secondary to missed diagnosis [4]. The following is a case presentation depicting the journey of clinical diagnosis and management of ML in the setting of a global pandemic where CDC support was unavailable.

## Case presentation

A 44-year-old male immigrant from South America is referred to the otolaryngology clinic for evaluation of nasal lesions and nasal airway obstruction. His history is remarkable for CL was diagnosed in 2016 after a two-year period of skin lesion development from an insect bite while working as a miner in Venezuela. There, he was treated with intravenous meglumine antimoniate over a six-month period in 2016 (68 doses), which resulted in full resolution of the lesions. Approximately six to twelve months after treatment completion, however, he developed sinus and nasal pain with obstructive nasal breathing and progressive disfigurement of his nose and face. He underwent repeat nasal biopsies in Venezuela in 2018; one of which was concerning for cutaneous carcinoma, but none revealed any microbial entity. He desired a second opinion and was lost to follow up until he relocated to Mexico in 2019 for work. There, a repeat biopsy revealed ML and he was subsequently treated with daily oral 150mg fluconazole for six months in January 2021 before finally presenting to our institution in July 2021. He was referred by the infectious disease team to the otolaryngology clinic. No leishmania speciation results were mentioned in the patient records. He noted that fluconazole had slowed the progression of the nasal symptoms but did not improve symptoms. 

**Figure 1:**
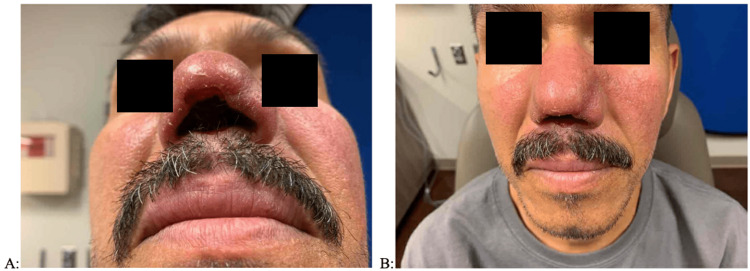
Pre-treatment examination, demonstrating erythema, scaling, and thickening of the face and nose skin, with the erosion of the nasal septum and columella, and compromise to the nasal architecture. (A) Base view. (B) Frontal view.

**Figure 2:**
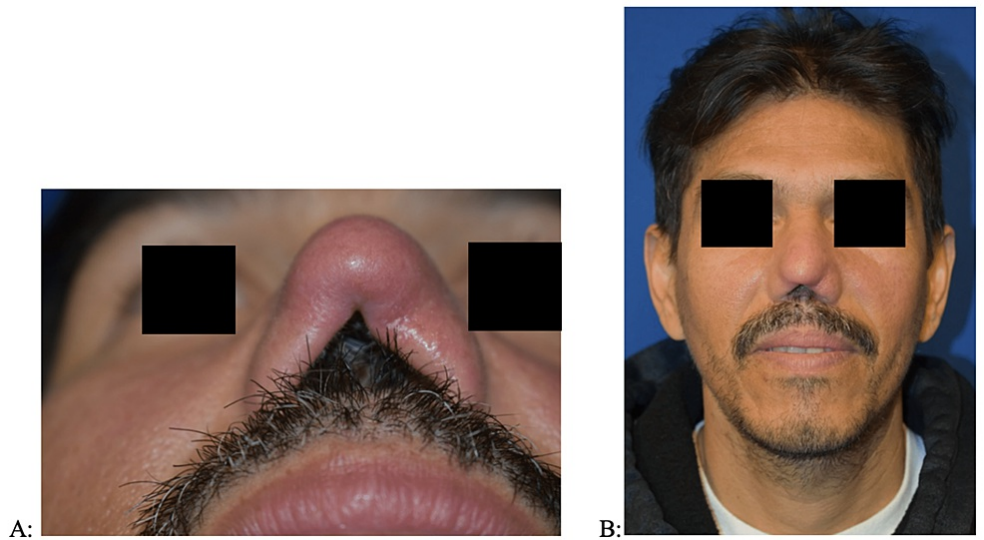
Post-treatment examination, demonstrating resolution of skin changes with healthier appearing nasal and facial skin, still with an absent nasal septum and disfigured nasal architecture. (A) Base view. (B) Frontal view.

Initial examination in the otolaryngology clinic was remarkable for completely absent columella and anterior septum with thickened and erythematous nasal skin envelope and nasal mucosa (Figures 1A, 1B). Additionally, there was an extension of erythema and thickening on the upper lip and bilateral cheeks. No oral lesions were present. A flexible laryngoscopy examination was not performed. Biopsies of the upper lip and nasal cavity demonstrated granulomatous inflammatory change, but no microorganisms were identified on gram staining or specialty staining (methanamine silver, giemsa, and acid-fast bacilli). He was treated by the infectious disease team with 28 days of oral miltefosine due to clinical suspicion of ML. At a three-month follow-up with ENT, the patient noted significant improvement in facial inflammation and breathing upon completion of miltefosine treatment. At the seven-month follow-up, examination demonstrated healing and regression of facial and nasal lesions (Figures 2A, 2B). At this point, treatment was deemed complete by infectious disease and thus suitable for nasal reconstruction. Due to overwhelming demand from the COVID-19 pandemic, The CDC was unable to process the sample for PCR testing, including intra-operative biopsies at the time of reconstruction. Therefore, diagnosis and management were based on a physical exam and the presence of granulomatous inflammation on biopsy. The patient has since undergone staged reconstruction of the nasal framework and has been asymptomatic since treatment with oral miltefosine. Of note, intra-operative findings demonstrated complete erosion of the cartilaginous and mucosal septum. Pharyngeal and laryngeal examinations were not performed. 

## Discussion

ML is an uncommon, but serious complication of untreated or poorly treated CL. The reported incidence is less than 5% and is considered indicative of severe or metastatic tegumentary leishmaniasis [9]. As demonstrated here, untreated ML can lead to facial disfigurement and functional impairment of the upper respiratory tract, and it can appear months to years after initial cutaneous presentation due to hematogenous or lymphatic spread [5].

The treatment strategy for CL and ML is widely variable in the literature due to the paucity of strong evidence to suggest a standard of treatment. Per the Infectious Disease Society of America guidelines, treatment should be individualized to take into consideration microbial species, area of endemic origin, and relevant host factors. Current medical management options for ML include parenteral pentavalent antimonials (sodium stibogluconate, meglumine antimoniate), amphotericin B, and oral miltefosine [6]. Unfortunately, each of these medication options can fall victim to resistance patterns, particularly when the mucosal spread is involved. Inpatient monitoring is warranted following emergency surgical intervention, or in the presence of laryngeal involvement to avoid possible airway complications [2].

In Latin America, the first-line management of ML is a pentavalent antimonial medication, commonly meglumine antimoniate or sodium stibogluconate [8,10]. These medications tend to be effective for CL in the absence of mucosal lesions. However, efficacy rates are lower in the presence of ML, particularly in the “mucosal belt” of Peru, Bolivia, Paraguay, and southern Brazil, where ML is highly endemic. On average, the treatment failure rate is endorsed at around 40% in these highly endemic regions [9,11]. Miltefosine has demonstrated effective cure rates in ML caused by the Vianna subgenus in Brazil, Bolivia, Ecuador, and Argentina, with cure rates upwards of 70% [12]. The FDA approved the use of oral miltefosine in 2014 as an alternative treatment for mucosal infection of L.V. braziliensis, along with cutaneous infection of L.V. braziliensis, L.V. guyanensis, and L.V. panamensis [12]. A number of case reports have also demonstrated the efficacy of treating ML and CL, including cases in the United States [12-15].

Despite the approval of miltefosine for refractory illness, there is little evidence to suggest it is superior in overall efficacy to pentavalent antimonial treatment. Multiple studies, including two prominent open-label randomized clinical trials, have demonstrated equivalent treatment rates between parenteral antimonial to oral miltefosine therapy. In 2018, Sampaio et al. performed an RCT comparing oral miltefosine and meglumine antimoniate for ML and found no significant difference in cure rates after four years when treating L.V. braziliensis in Brazil [8]. The average cure rate for both treatments was around 66%, which is consistent with the averages across the literature. However, within the first 90 days of treatment, the miltefosine group had a cure probability over two times greater than those treated with memantine. Patients were also less fearful and more tolerant of the milder side effects of miltefosine, suggesting that miltefosine may allow faster re-epithelialization of mucosal lesions and with less concern for unwanted side effects [8]. The most commonly presented side effects of pentavalent antimonial medications are myalgias/arthralgias, transaminase and pancreatic amylase elevation, and cardiac arrhythmia. Pentavalent antimonials are also contraindicated in renal insufficiency [8]. In comparison, the most commonly presented side effects of miltefosine are limited to gastrointestinal effects of nausea, vomiting, diarrhea, and anorexia [8]. Similar findings were seen in a more recent 2021 RCT comparing miltefosine and sodium stibogluconate in the treatment of L.V. braziliensis in Brazil. This trial found no statistically significant difference between the two medications’ curative potential. However, a statistically significant increase in unwanted side effects from the sodium stibogluconate treatment was present, which may result in more frequent interruptions in treatment and subsequent prolongation of treatment duration [11]. Cochrane review of systemic therapies for cutaneous and mucocutaneous leishmaniasis concluded that meglumine antimoniate and miltefosine were the most likely systemic medications to have increased rates of cure, but that evidence was mostly low to moderate in quality [16]. A phase II RCT by Francesconi et al. in 2018 found cure rates of meglumine antimoniate at 53%-70% and miltefosine at 54%-72% [4].

After miltefosine and pentavalent antimony, the last remaining treatment option for ML is Amphotericin B, which is typically reserved for refractory cases to meglumine antimoniate and miltefosine treatment [6,13]. This medication is known for a more severe side effect profile, specifically nephrotoxicity, anemia, and electrolyte disturbance [2]. Oral azole therapy has shown no significant efficacy against new world leishmania infections and ML [2]. This is consistent with our patient's case, as there was no improvement in oral fluconazole. The decision to treat our patient with Oral Miltefosine was rooted in the IDSA treatment guidelines for Leishmaniasis [2]. Pentavalent antinomy is not available at our institution and liposomal amphotericin B was avoided due to the need for central line placement and a more severe side effect profile. 

Variability in resistance patterns is currently a major obstacle in the pursuit of pinpointing a reliable management strategy for ML. For example, miltefosine has been shown to have acceptable treatment rates for L.V. braziliensis in Bolivia and Brazil (88% and 75%, respectively), but its efficacy is proven considerably worse when used in Guatemalan cases of L.V. braziliensis (45%) [7,10,17]. This obstacle to effective management is simply due to the fact that the mechanism of resistance patterns is poorly understood at the moment. However, we are aware that resistance to pentavalent antimonial agents follows similar trends throughout endemic regions. For instance, a 2008 meta-analysis found treatment efficacy in endemic regions of Brazil is significantly worse than in Columbia for CL (71% vs 91%, respectively), and that average cure rates for new world CL were about 76% overall [18]. Cases with mucosal lesions were found to have even lower treatment efficacy. There is currently no literature that compares resistance patterns in endemic areas for ML because the majority of the literature has aimed their focus on resistance patterns in CL. This is likely due to several factors. First, the percentage of mucosal metastasis of CL is low. Second, the low socioeconomic status of endemic ML areas affects access to healthcare. This leads to inconsistencies in follow-up, and ultimately an incomplete picture of the optimal management for these patients [18]. 

This review of the current literature demonstrates that in the face of growing resistance, miltefosine and pentavalent antimonials have nearly equivalent treatment rates, and consideration for treatment should focus more on patient-centric variables such as comorbidities and access to healthcare. Given the geography-based resistance patterns mentioned above, one patient-centric variable that the treating provider may consider is the patient’s country of emigration. Although the current situation of treatment is one of uncertainty, the future looks bright. De Souza states that new literature is emerging on the development of novel treatment strategies for resistant cases. For instance, it is thought that there is a potential for efficacy in treating resistant strains of L.V. panamensis with combination therapy of posaconazole and miltefosine [19].

In this case presentation, the patient did not have a positive culture and no microbial organisms present on histopathology. An additional hurdle in the diagnosis of this patient was that DNA-based assay and molecular analysis via the CDC were not available due to the overwhelming burden of the COVID-19 pandemic on the CDC. However, suspicion for ML was raised when histopathology showed a granulomatous inflammatory change in the setting of prior a CL infection. One other report describes ML masquerading as idiopathic midline granulomatous disease. In this case report, a 44-year-old female was treated for necrotizing granulomatous disease of the skin and nose for five years when the initial septal biopsy demonstrated necrotizing granulomatous disease without microorganisms. Repeat biopsy with appropriate staining years later revealed microorganisms confirmed to be L.V. panamensis which was successfully treated with two courses of liposomal amphotericin B [14]. The female described did not have a prior diagnosis of CL, as her cutaneous lesions were attributed to the granulomatous disease.

Lastly, this case presentation is an example of the importance of a multidisciplinary approach to treating ML. Otolaryngology evaluation in patients with ML is recommended for mucosal examination and endoscopic evaluation of the nasal cavity, pharynx, and larynx [2,6]. Additionally, immediate surgical intervention may be necessary for the setting of airway obstruction secondary to laryngeal lesions. Consultation with infectious disease is prudent for the expertise in management based on speciation and region-specific resistance patterns. Once a patient is considered cured with serially negative biopsies and clinical evidence of lesion regression, reconstructive surgery can be undertaken.

## Conclusions

Leishmaniasis is uncommon in the United States. CL can metastasize to the mucous membranes of the upper aerodigestive tract and can be difficult to manage. ML has the additional implication of compromising facial aesthetics as well as respiratory integrity. Here we present a case of ML, without confirmation from a CDC reference laboratory, and the treatment. A clinical history of cutaneous disease with mucosal biopsy showing chronic granulomatous inflammation can be a strong indicator of metastatic disease in the absence of microbial isolation. We also demonstrate a multi-disciplinary treatment strategy, driven by infectious disease and otolaryngology.
